# Supplementary material for: Bridging the gap in medical education: comparing analysis of light microscopy and virtual microscopy in histology
Source: PeerJ. 2024 Jul 15;12:e17695. doi: 10.7717/peerj.17695 (PMC11257044; doi:10.7717/peerj.17695)
Supplement: Supplemental Information 2 [file peerj-12-17695-s002.docx]

| **Survey question in Turkish** | **Survey question in English** |
| --- | --- |
| A. Etkinlik [Işık mikroskop kullanarak zamanımı etkili bir şekilde yönetebildim] | A. Activity [I was able to manage my time effectively using a light microscope] |
| A. Etkinlik [Işık mikroskop dersin amaç ve öğrenim hedefleri doğrultusunda etkilidir] | A. Activity [Light microscope is effective in line with the aims and learning objectives of the lesson] |
| A. Etkinlik [Işık mikroskop kullanımı konuların akılda kalıcılığını artırdı] | A. Activity [The use of light microscope increased the memorability of the topics] |
| A. Etkinlik [Işık mikroskop konuyu öğrenmeme yardımcı oldu] | A. Activity. [Light microscope helped me learn the subject] |
| A. Etkinlik [Işık mikroskopinin ders saatleri dışında kullanımı histoloji pratik sınavına hazırlanırken bilgilere erişimimi kolaylaştırdı.] | A. Activity[The use of light microscopy outside of class hours facilitated my access to information while preparing for the histology practical exam]. |
| A. Etkinlik [Derste Işık mikroskop kullanımı histoloji konularına merakımı ve motivasyonumu artırdı.] | A. Activity [Using the light microscope in class increased my curiosity and motivation for histology topics]. |
| A. Etkinlik [Sanal mikroskop kullanarak zamanımı etkili bir şekilde yönetebildim] | A. Activity [Using the virtual microscope allowed me to manage my time effectively] |
| A. Etkinlik [Sanal mikroskop dersin amaç ve öğrenim hedefleri doğrultusunda etkilidir] | A. Activity [Virtual microscope is effective in line with the aims and learning objectives of the course] |
| A. Etkinlik [Sanal mikroskop kullanımı konuların akılda kalıcılığını artırdı.] | A. Activity [The use of virtual microscope increased the retention of the topics]. |
| A. Etkinlik [Sanal mikroskop konuyu öğrenmeme yardımcı oldu] | A. Activity [Virtual microscope helped me learn the subject] |
| A. Etkinlik [Sanal mikroskopinin ders saatleri dışında kullanımı histoloji pratik sınavına hazırlanırken bilgilere erişimimi kolaylaştırdı.] | A. Activity [Using the virtual microscope outside of class hours facilitated my access to information while preparing for the histology practical exam]. |
| A. Etkinlik [Derste sanal mikroskop kullanımı histoloji konularına merakımı ve motivasyonumu artırdı.] | A. Activity [Using the virtual microscope in class increased my curiosity and motivation for histology topics]. |
| B. Görüntü Kalitesi [Işık mikroskop, dokuları ayrıntılı biçimde büyüterek incelememi kolaylaştırdı] | B. Image Quality [The light microscope made it easier for me to examine the tissues by magnifying them in detail] |
| B. Görüntü Kalitesi [Sanal mikroskop, dokuları ayrıntılı biçimde büyüterek incelememi kolaylaştırdı] | B. Image Quality [The virtual microscope facilitated my examination by magnifying the tissues in detail] |
| C. Kullanım Kolaylığı [Işık mikroskop kullanımı için talimatlar açık ve netti] | C. Ease of Use [Instructions for using the light microscope were clear and concise] |
| C. Kullanım Kolaylığı [Işık mikroskop kullanımı kolaydı] | C. Ease of Use [The light microscope was easy to use] |
| C. Kullanım Kolaylığı [Işık mikroskop ile görüntüleri kolayca geziyorum] | C. Ease of Use [I easily navigate the images with the light microscope] |
| C. Kullanım Kolaylığı [Sanal mikroskop kullanımı için talimatlar açık ve netti] | C. Ease of Use [Instructions for using the virtual microscope were clear and concise] |
| C. Kullanım Kolaylığı [Sanal mikroskop kullanımı kolaydı] | C. Ease of Use [Virtual microscope was easy to use] |
| C. Kullanım Kolaylığı [Sanal mikroskop ile görüntüleri kolayca geziyorum] | C. Ease of Use [I can easily navigate the images with the virtual microscope] |
| D. İşbirlikli Kullanım [Işık mikroskobi, diğer öğrencilerle işbirliği yapmamı sağladı] | D. Collaborative Use [Light microscopy allowed me to collaborate with other students] |
| D. İşbirlikli Kullanım [Işık mikroskobu planlanan ders saati dışında ve istediğim mekanda kullanabilirim] | D. Collaborative Use [I can use the light microscope outside of the planned class hours and in any place I want] |
| D. İşbirlikli Kullanım [Sanal mikroskobi, diğer öğrencilerle işbirliği yapmamı sağladı] | D. Collaborative Use [Virtual microscopy allowed me to collaborate with other students] |
| D. İşbirlikli Kullanım [Sanal mikroskobu planlanan ders saati dışında ve istediğim mekanda kullanabilirim] | D. Collaborative Use [I can use the virtual microscope outside of the planned class hours and at any place I want] |
| E. Teknik Destek Alma [Işık mikroskop ders saati içerisinde kullanırken, kesitlere eğiticinin müdahalesi daha kolay oldu.] | E. Receiving Technical Support [When using the light microscope during the lesson, it was easier for the instructor to intervene in the sections]. |
| E. Teknik Destek Alma [Işık mikroskop ile görüntüleri gezerken sorun yaşadım] | E. Receiving Technical Support [I had problems while viewing the images with the light microscope] |
| E. Teknik Destek Alma [Işık mikroskop kullanımı sırasında karşılaşılan teknik sorunlara hemen müdahale edilebilir] | E. Getting Technical Support [Technical problems encountered during the use of the light microscope can be intervened immediately] |
| E. Teknik Destek Alma [Işık mikroskop kullanımında eğitici desteğine daha çok ihtiyaç duyulmaktadır.] | E. Getting Technical Support [Instructor support is needed more in the use of light microscope]. |
| E. Teknik Destek Alma [Sanal mikroskop ders saati içerisinde kullanırken, kesitlere eğiticinin müdahalesi daha kolay oldu.] | E. Receiving Technical Support [When using the virtual microscope during the lesson, it was easier for the instructor to intervene in the sections]. |
| E. Teknik Destek Alma [Sanal mikroskop ile görüntüleri gezerken sorun yaşadım] | E. Receiving Technical Support [I had problems navigating the images with the virtual microscope] |
| E. Teknik Destek Alma [Sanal mikroskop kullanımı sırasında karşılaşılan teknik sorunlara hemen müdahale edilebilir] | E. Getting Technical Support [Technical problems encountered during the use of the virtual microscope can be intervened immediately] |
| E. Teknik Destek Alma [Sanal mikroskop kullanımında eğitici desteğine daha çok ihtiyaç duyulmaktadır.] | E. Receiving Technical Support [Instructor support is needed more in the use of virtual microscope]. |
| F. Tercih/ eğlence durumu [Histoloji dersinde öğrenim aracı olarak Işık mikroskobu tercih ederim] | F. Preference / entertainment status [I prefer light microscope as a learning tool in histology course] |
| F. Tercih/ eğlence durumu [Işık mikroskobu histoloji eğitiminde kullanmak eğlencelidir.] | F. Preference/fun status [It is fun to use the light microscope in histology education]. |
| F. Tercih/ eğlence durumu [Histoloji dersinde öğrenim aracı olarak sanal mikroskobu tercih ederim] | F. Preference/ recreational status [I prefer virtual microscope as a learning tool in histology course] |
| F. Tercih/ eğlence durumu [Sanal mikroskobu histoloji eğitiminde kullanmak eğlencelidir.] | F. Preference/fun status [It is fun to use virtual microscopy in histology education]. |
| 19. Sanal mikroskopi ve Işık mikroskopinin histoloji eğitiminize katkılarını ve geliştirilmesi gereken yönleri ile ilgili her türlü ek geri bildirimi yazınız. | 19. Please write down any additional feedback about the contribution of Virtual microscopy and Light microscopy to your histology education and any aspects that need to be improved. |
|  |  |
| **Turkish Questionnaire Answers (Likert Type)** | **English Questionnaire Answers (Likert Type)** |
| 5. Kesinlikle katılıyorum | 5. Strongly agree |
| 4. Katılıyorum | 4. I agree |
| 3. Kararsızım | 3. Undecided |
| 2. Katılmıyorum | 2. I disagree |
| 1. Kesinlikle katılmıyorum | 1. Strongly disagree |
